# Association between systemic inflammation response index and osteoporosis in perimenopausal and postmenopausal women: A retrospective observational study

**DOI:** 10.1097/MD.0000000000049106

**Published:** 2026-06-12

**Authors:** Xiaoning Wang, Doufei Shi

**Affiliations:** a Department of Geriatrics, Binzhou Medical University Hospital, Binzhou, Shandong, China.

**Keywords:** osteoporosis, perimenopausal and postmenopausal women, predictive value, systemic inflammation response index (SIRI)

## Abstract

This study aimed to analyze the risk factors of osteoporosis in perimenopausal and postmenopausal women, especially the correlation between inflammatory indicators and osteoporosis and construct a risk prediction model for osteoporosis. A total of 299 perimenopausal and postmenopausal women (40–60 years old) were enrolled from September 2023 to September 2025, who underwent physical examination or hospitalization at the Affiliated Hospital of Binzhou Medical University. They were stratified into 3 groups based on lumbar spine (L1–L4) bone mineral density measurements: normal bone mass group (n = 112), osteopenia group (n = 105), and osteoporosis group (n = 82). General clinical data, laboratory indicators, and composite inflammatory markers – including the systemic inflammation response index (SIRI), systemic immune-inflammation index, and neutrophil-to-lymphocyte ratio – were collected. Spearman correlation analysis, univariate/multivariate logistic regression analyses, and LASSO regression analysis were performed. An osteoporosis risk nomogram was constructed, and the clinical assessment utility for evaluating the association with osteoporosis was assessed using the receiver operating characteristic curve (ROC). Significant differences in age, body mass index, diabetes history, neutrophils, and lymphocytes were noted among the 3 groups (all *P* < .05). SIRI was negatively correlated with bone mineral density (*r* = −0.203, *P* < .001) and positively correlated with osteoporosis (*r* = 0.273, *P* < .001). Multivariate logistic regression confirmed SIRI as an independent risk factor for osteoporosis after adjusting for confounding variables (OR = 5.841, 95% CI: 2.721–12.541, *P* < .001). The area under the receiver operating characteristic curve of SIRI for predicting osteoporosis was 0.677. Meanwhile, the combined model (SIRI + diabetes history + age + low-density lipoprotein cholesterol + history of frequent falls) achieved a significantly higher area under the curve of 0.802 (*P* < .001). SIRI is significantly associated with osteoporosis in perimenopausal and postmenopausal women, providing potential clinical reference value for risk assessment. The combined assessment model incorporating age, low density lipoprotein cholesterol, diabetes history, and history of frequent falls can enhance clinical assessment utility for evaluating the association with osteoporosis.

## 1. Introduction

Osteoporosis is a systemic skeletal disorder characterized by reduced bone mass and deteriorated bone microarchitecture, leading to increased bone fragility and elevated fracture risk.^[1]^ According to the national epidemiological survey of osteoporosis in China, the prevalence of osteoporosis is 3.2% and the rate of low bone mass is 32.9% among individuals aged 40 to 49 years. Among those aged over 50 years, the prevalence of osteoporosis reaches 19.2% and the rate of low bone mass is 46.4%.^[2]^ Epidemiological projections indicate that the annual global incidence of osteoporotic fractures will surge from 6.9 million in 2020 to 16.2 million in 2040, with an average annual growth rate of 8.7%, and the related economic burden will increase by 121%.^[3]^ Notably, osteoporosis is characterized by an insidious onset and the absence of specific early clinical symptoms. Most patients are only diagnosed after a fragility fracture occurs, which leads to the loss of the optimal timing for early intervention. Therefore, early prevention is the core strategy for the clinical management of osteoporosis and related low-energy fractures, and preventive interventions mainly include healthy lifestyles and sufficient nutritional intake.^[4]^

In recent years, inflammation has emerged as a key factor influencing bone health. Accumulating evidence suggests that chronic low-grade inflammation is closely associated with various metabolic diseases and may play a pivotal role in bone metabolism.^[5]^ Novel composite inflammatory indices have been developed based on routine hematological parameters. These include the systemic inflammation response index (SIRI), systemic immune-inflammation index (SII), and neutrophil-to-lymphocyte ratio (NLR). Among these indices, SIRI is calculated based on 3 core immune cells involved in inflammatory regulation: monocytes, neutrophils, and lymphocytes. Increasing studies have demonstrated that these novel inflammatory markers can effectively reflect the body’s inflammatory and immune status. They can also predict the occurrence and progression of various diseases,^[6–9]^ such as cardiovascular diseases, metabolic syndrome, diabetes, and cancer. However, the specific molecular mechanism underlying inflammation-mediated osteoporosis pathogenesis remains poorly understood. Existing evidence suggests that immune cells may participate in the occurrence and development of osteoporosis by directly or indirectly regulating the physiological activities of bone cells.^[10]^

As a novel inflammatory biomarker, SIRI has prominent clinical advantages including easy accessibility, favorable reproducibility, and low cost in routine clinical settings. Compared with single hematological indicators (e.g., monocyte and lymphocyte), SIRI can reflect the systemic immune-inflammatory state in a more stable and comprehensive manner. Despite the above advantages, the correlation between SIRI and the risk of osteoporosis in perimenopausal and postmenopausal women has not been fully elucidated.

We hypothesized that elevated SIRI levels are independently associated with an increased risk of osteoporosis, and that SIRI can serve as a reliable predictive biomarker for osteoporosis in perimenopausal and postmenopausal women.

Accordingly, the present study was designed to identify the risk factors of osteoporosis, analyze the correlation between SIRI and osteoporosis, and evaluate the clinical reference value of SIRI for osteoporosis in perimenopausal and postmenopausal women. The results of this study may provide a scientific basis and practical reference for the early identification and individualized prevention of osteoporosis in this high-risk population.

## 2. Materials and methods

### 2.1. Study participants

A total of 299 perimenopausal and postmenopausal women aged 40 to 60 years were included in this study. The sample size was determined based on the prevalence of osteoporosis in perimenopausal and postmenopausal women and the expected effect size of SIRI. These subjects visited the Affiliated Hospital of Binzhou Medical University for hospitalization or physical examination between September 2023 and September 2025. Inclusion criteria: completed bone mineral density (BMD) examination with complete general clinical data; perimenopausal or postmenopausal women aged 40 to 60 years; met the World Health Organization diagnostic criteria for osteoporosis; no history of hormone replacement therapy in the past 6 months. Exclusion criteria: secondary osteoporosis (e.g., hyperparathyroidism and rheumatoid arthritis); incomplete medical records or clinical data; use of drugs affecting bone metabolism (e.g., calcium carbonate, vitamin D, bisphosphonates, and glucocorticoids) or immunosuppressants (e.g., cyclosporine) in the past 3 months; complications of consumptive diseases (e.g., tuberculosis and malignant tumors); and history of acute infections (e.g., pneumonia and urinary tract infections) in the past year. The clinical data and laboratory test results of the participants were strictly confidential and not disclosed to any third parties. All participants voluntarily participated in this study. This study was approved by the Ethics Committee of the First Clinical Medical College, Affiliated Hospital of Binzhou Medical University (approval number: KYLL-347). Due to the retrospective nature of this study, the ethics committee waived the requirement for obtaining informed consent from the participants.

### 2.2. Data collection

Relevant data were extracted from the electronic medical record system of the affiliated hospital of Binzhou medical university. General clinical data included age, height, weight, body mass index (BMI), diabetes history, smoking history, drinking history, age at menarche, exercise habits, frequent fall history, coronary heart disease history, and previous fracture history. Laboratory indicators measured at admission included complete blood count and lipid profiles. BMD of the lumbar spine (L1–L4) was measured using dual-energy X-ray absorptiometry. The T-score was calculated based on the average BMD of the L1–L4 segments. Daily quality control of the dual-energy X-ray absorptiometry instrument was performed by dedicated technicians to ensure the accuracy of measurements. The BMD values referenced in this study refer to T-scores.

### 2.3. Definitions of relevant indicators

#### 2.3.1. Calculation of indicators

BMI = weight(kg)/ height^2^ (m^2^);
SIRI = (neutrophil count, 10^9^/L) × (monocyte count, 10^9^/L)/(lymphocyte count, 10^9^/L);
NLR = (neutrophil count, 10^9^/L)/(lymphocyte count, 10^9^/L);
SII = (platelet count, 10^9^/L) × (neutrophil count, 10^9^/L)/(lymphocyte count, 10^9^/L).

#### 2.3.2. Definition of clinical variables

Diabetes history was defined as a diagnosis of diabetes mellitus established by a secondary or higher-level hospital in accordance with the 1999 World Health Organization diagnostic criteria, or fasting blood glucose ≥ 7.0 mmol/L combined with glycated hemoglobin ≥ 6.5% on admission. Cerebrovascular disease history was defined as a diagnosis of cerebral infarction, cerebral hemorrhage, or transient ischemic attack confirmed by cranial computed tomography or magnetic resonance imaging. Exercise habits were defined as engagement in moderate-intensity exercise for ≥ 30 minutes per session, ≥3 times per week, for at least 1 consecutive year. Smoking history was defined as current smoking with a cumulative duration of ≥ 6 months; nonsmokers comprised lifelong nonsmokers and former smokers who had quit smoking for ≥ 6 months at enrollment. Drinking history was defined as current regular alcohol consumption of ≥ 1 occasion per week for ≥ 6 consecutive months; nondrinkers were defined as individuals who never drank alcohol or drank only occasionally without meeting the aforementioned criteria.

### 2.4. Statistical methods

SPSS 26.0 software was used for statistical analysis. Normally distributed continuous data were expressed as mean ± standard deviation (x ± s). Comparisons between groups were analyzed using one-way analysis of variance. Non-normally distributed continuous data were described as median and interquartile range [M(P25, P75)], Intergroup comparisons were performed using the Kruskal–Wallis H test, and post hoc pairwise comparisons were conducted with the bonferroni correction. Categorical data were expressed as frequency and percentage [n(%)], and comparisons between groups were performed using the χ^2^ test. Spearman correlation analysis was used to explore the correlations between variables. Univariate logistic regression, multivariate logistic regression, and LASSO regression models were used to screen the risk and protective factors of osteoporosis in perimenopausal and postmenopausal women. Receiver operating characteristic curves (ROCs) were used to determine the clinical assessment value and optimal cutoff value of SIRI for assessing the association with osteoporosis. A two-tailed *P* < .05 was considered statistically significant.

## 3. Results

### 3.1. Comparison of general clinical data among groups with different bone mass status

A total of 299 perimenopausal and postmenopausal women were included in this study. They were stratified into 3 groups according to lumbar spine BMD: normal bone mass group (n = 112), osteopenia group (n = 105), and osteoporosis group (n = 82). Comparisons of general clinical data and laboratory indicators among the 3 groups are presented in Table 1. Significant differences were observed in neutrophil, platelet, lymphocyte, monocyte, NLR, SIRI, high density lipoprotein cholesterol (HDL-C), low density lipoprotein cholesterol (LDL-C), total cholesterol (TC), age, BMI, diabetes history, education level, exercise habits, frequent fall history, and fracture history among the 3 groups (all *P* < .05).

**Table 1:** Comparison of general data among the 3 groups.

	Normal bone mass group(T1 = 112)	Osteopenia group (n = 105)	Osteoporosis group(n = 82)	χ^2^/*H*/*F*	*P*
Age	52 (48–56)	57 (54–59)***	57 (53–59)***	*H* = 45.406	<.001
BMI	24.70 (22.65–27.15)	23.60 (21.72–25.40)*	22.60 (21.12–24.04)***	*H* = 20.624	<.001
Diabetes history					
Yes	9 (8.0%)	19 (18.1%)	30 (36.6%)	χ^2^ = 28.855	<.001
No	103 (92.0%)	86 (81.9%)	52 (63.4%)		
Hypertension history					
Yes	30 (26.8%)	31 (29.5%)	26 (31.7%)	χ^2^ = 0.570	.752
No	82 (73.2%)	74 (70.5%)	56 (68.3%)		
College education or Above					
Yes	29 (25.9%)	10 (9.5%)	10 (12.2%)	χ^2^ = 12.047	.002
No	83 (74.1%)	95 (90.5%)	72 (87.8%)		
Smoking history					
Yes	7 (6.3%)	6 (5.7%)	3 (3.7%)	χ^2^ = 0.716	.699
No	105 (93.8%)	99 (94.3%)	79 (96.3%)		
Drinking history					
Yes	3 (2.7%)	4 (3.8%)	2 (2.4%)	χ^2^ = 0.354	.836
No	109 (97.3%)	101 (96.2%)	80 (97.6%)		
Number of pregnancies (>2)					
Yes	54 (48.2%)	59 (56.2%)	36 (43.9%)	χ^2^ = 2.969	.227
No	58 (51.8%)	46 (43.8%)	46 (56.1%)		
Age at Menarche (>14 years)					
Yes	41 (36.6%)	44 (41.9%)	27 (32.9%)	χ^2^ = 1.640	.441
No	71 (63.4%)	61 (58.1%)	55 (67.1%)		
Exercise habits					
Yes	40 (35.7%)	25 (23.8%)	14 (17.1%)	χ^2^ = 9.030	.011
No	72 (64.3%)	80 (76.2%)	68 (82.9%)		
Frequent fall history					
Yes	14 (12.5%)	18 (17.1%)	32 (39.0%)	χ^2^ = 21.545	<.001
No	98 (87.5%)	87 (82.9%)	50 (61.0%)		
Chronic liver/kidney disease					
Yes	32 (28.6%)	24 (22.9%)	18 (22.0%)	χ^2^ = 1.425	.490
No	80 (71.4%)	81 (77.1%)	64 (78.0%)		
Previous fracture history					
Yes	13 (11.6%)	22 (21.0%)	25 (30.5%)	χ^2^ = 10.600	.005
No	99 (88.4%)	83 (79.0%)	57 (69.5%)		
Cerebrovascular disease history					
Yes	10 (8.9%)	14 (13.3%)	11 (13.4%)	χ^2^ = 1.377	.513
No	102 (91.1%)	91 (86.7%)	71 (86.6%)		
Coronary heart disease history					
Yes	9 (8.0%)	7 (6.7%)	6 (7.3%)	χ^2^ = 0.149	.928
No	103 (92.0%)	98 (93.3%)	76 (92.7%)		
Lymphocyte(10^9^/L)	2.10 (1.70–2.50)	2.00 (1.60–2.40)	1.80 (1.4–2.3)**	*H* = 9.660	.008
Monocyte(10^9^/L)	0.30 (0.20–0.30)	0.30 (0.20–0.40)	0.30 (0.20–0.40)*,#	*H* = 9.494	.009
Neutrophil(10^9^/L)	3.00 (2.45–3.50)	2.70 (2.30–3.40)	3.40 (2.60–4.40)##	*H* = 12.950	.002
Platelet(10^9^/L)	257.76 ± 60.60	245.21 ± 55.75	236.13 ± 60.74	*F* = 3.296	.038
NLR	1.43 (1.15–1.86)	1.32 (1.10– 1.76)	1.76 (1.30– 2.64)##,**	*H* = 15.588	<.001
SII	357.42 (274.45– 487.95)	347.56 (271.36–470.00)	395.71 (286.00–646.15)	*H* = 4.198	.123
SIRI	0.37 (0.27–0.57)	0.38 (0.26–0.54)	0.58 (0.35–1.06)***,###	*H* = 22.200	<.001
LDL-C(mmol/L)	3.35 ± 0.87	3.30 ± 0.94	2.94 ± 0.93**,##	*F* = 5.522	.008
HDL-C(mmol/L)	1.46 (1.23–1.70)	1.51 (1.30–1.68)	1.39 (1.16–1.57)#	*H* = 7.049	.029
Triglyceride(mmol/L)	1.25 (0.90–1.81)	1.19 (0.93–1.67)	1.18 (0.84–1.64)	*H* = 1.328	.515
TC(mmol/L)	5.16 ± 0.89	5.40 ± 1.03	5.54 ± 0.50*	*F* = 4.369	.046

Post hoc pairwise comparisons among the 3 groups were performed using the Bonferroni method.

BMI = body mass index, HDL-C = high density lipoprotein cholesterol, LDL-C = low density lipoprotein cholesterol, NLR = neutrophil to lymphocyte ratio, SII = systemic immune-inflammation index, SIRI = systemic inflammation response index, TC = total cholesterol.

* *P* < .05, **P < .01, ***P < .001 versus normal bone mass group.

# *P* < .05, ##*P* < .01, ###*P* < .001 versus osteopenia group.

Further post hoc pairwise comparisons were performed. Compared with the normal bone mass group, the osteoporosis group exhibited higher age, NLR, and SIRI, as well as lower BMI and LDL-C (all *P* < .05). Compared with the osteopenia group, the osteoporosis group had higher neutrophil and lower HDL-C (all *P* < .05). Additionally, the osteoporosis group had lower lymphocyte and higher TC than the normal bone mass group (all *P* < .05).

### 3.2. Analysis of the correlation between SIRI and various indicators

Spearman correlation analysis was performed to investigate the associations between SIRI, various clinical indicators, and BMD (Table 2). The results demonstrated a negative correlation between SIRI and BMD (*r* = −0.203, *P* < .001), and a positive correlation between SIRI and NLR, SII, diabetes history, as well as cerebrovascular disease history.

**Table 2:** Spearman correlation analysis of SIRI with various indicators and BMD.

Indicator 1	Indicator 2	*r*	*P*	Indicator 1	Indicator 2	*r*	*P*
SIRI	Platelet	0.020	.734		BMI	−0.010	.868
	TC	−0.071	.218		HDL-C	−0.102	.079
	Triglyceride	−0.037	.524		LDL-C	−0.110	.058
	Age	−0.042	.464		Drinking history	0.048	.407
	NLR	0.695	<**.001**		SII	0.130	**.025**
	Hypertension history	0.072	.212		Smoking history	0.037	.529
	Diabetes history	0.208	<**.001**	SIRI	Fracture history	0.072	.216
	Exercise habits	0.028	.630		Coronary heart disease history	0.016	.786
	College education or above	0.033	.566		Cerebrovascular disease history	0.115	**.047**
	Chronic liver/kidney disease history	0.019	.743		Frequent fall history	0.080	.167
	BMD	−0.203	<**.001**				

Bold values indicate *P* < .05.

BMD = bone mineral density, BMI = body mass index, HDL-C = high density lipoprotein cholesterol, LDL-C = low density lipoprotein cholesterol, NLR = neutrophil to lymphocyte ratio, SIRI = systemic inflammation response index, TC = total cholesterol.

### 3.3. Results of univariate logistic regression analysis

The presence of osteoporosis was defined as the dependent variable (yes = 1, no = 0), and indicators with *P* < .05 in inter-group comparisons were selected as independent variables. Categorical variables (e.g., diabetes history, college education or above, exercise habits, frequent fall history, and fracture history) were assigned values (Table 3). Subsequently, univariate logistic regression analysis was performed. For monocyte, the raw data had insufficient variability, which resulted in overly wide confidence intervals. Therefore, the data were uniformly expanded 10-fold to improve variable dispersion prior to inclusion in the analysis.^[11]^

**Table 3:** Assignment explanation.

Variable	Assignment explanation
Diabetes (Yes/No)	No = 0, Yes = 1
College education or above	No = 0, Yes = 1
Exercise habits (Yes/No)	No = 0, Yes = 1
Frequent falls (Yes/No)	No = 0, Yes = 1
Previous fracture (Yes/No)	No = 0, Yes = 1
Number of pregnancies (>2 times)	No = 0, Yes = 1
Age at Menarche (>14 yrs)	No = 0, Yes = 1
Cerebrovascular disease (Yes/No)	No = 0, Yes = 1

As presented in Table 4, age, BMI, LDL-C, TC, lymphocyte, monocyte, neutrophil, NLR, SIRI, diabetes history, exercise habits, frequent fall history, and fracture history were significantly associated with osteoporosis (all *P* < .05). Among these variables: age, TC, monocyte, neutrophil, NLR, SIRI, diabetes history, frequent fall history, and fracture history were identified as risk factors for osteoporosis; BMI, LDL-C, lymphocyte, and exercise habits were protective factors for osteoporosis. No significant associations were observed between other variables and osteoporosis (all *P* > .05).

**Table 4:** Univariate logistic regression analysis.

Variable	Regression coefficient	Standard error	*P*	OR	95% Confidence interval
Age	0.075	0.029	.010	1.078	1.018–1.142
BMI	−0.164	0.049	.001	0.849	0.772–0.934
LDL-C	−0.483	0.152	.001	0.617	0.458–0.830
HDL-C	0.029	0.033	.386	1.029	0.964–1.098
TC	0.320	0.146	.029	1.377	1.033–1.834
Lymphocyte	−0.622	0.227	.006	0.537	0.344–0.838
Monocyte	0.388	0.114	.001	5.156	1.178–1.843
Neutrophil	0.344	0.099	.001	1.411	1.162–1.713
NLR	0.541	0.146	<.001	1.717	1.290–2.286
SIRI	1.478	0.337	<.001	4.383	2.263–8.488
Diabetes history	1.359	0.306	<.001	3.894	2.138–7.092
College education or above	−0.456	0.381	.231	0.634	0.300–1.338
Exercise habits	−0.731	0.329	.026	0.481	0.253–0.917
Frequent fall history	1.308	0.296	<.001	3.700	2.069–6.616
Previous fracture history	0.824	0.303	.006	2.260	1.260–4.128

BMI = body mass index, HDL-C = high density lipoprotein cholesterol, LDL-C = low density lipoprotein cholesterol, NLR = neutrophil to lymphocyte ratio, SIRI = systemic inflammation response index, TC = total cholesterol.

### 3.4. Multivariate logistic regression analysis of SIRI, NLR, and osteoporosis

To further verify the independent association between SIRI and osteoporosis, multivariate logistic regression analysis was performed. Three models were constructed for this analysis: an unadjusted model, Model I (adjusted for age, exercise habits, smoking history, drinking history, and BMI), and Model II. Model II was further adjusted for diabetes history, coronary heart disease history, chronic liver/kidney disease history, cerebrovascular disease history, hypertension history, number of pregnancies, age at menarche, TC, HDL-C, and LDL-C on the basis of Model I.

As shown in Table 5, in the unadjusted model, SIRI was significantly associated with an increased risk of osteoporosis (OR = 4.383, 95% CI: 2.263–8.488, *P* < .001). In Model I, after adjusting for basic confounding factors (age, exercise habits, smoking history, drinking history, and BMI), SIRI remained a significant risk factor for osteoporosis (OR = 4.803, 95% CI: 2.356–9.791, *P* < .001). This indicates that the predictive effect of SIRI on osteoporosis was not attenuated by these confounding factors. In Model II, after further adjusting for additional confounding variables, SIRI still acted as an independent risk factor for osteoporosis (OR = 5.841, 95% CI: 2.721–12.541, *P* < .001). The OR values remained stable before and after adjustment, confirming the robustness of the association between SIRI and osteoporosis. For the control indicator NLR, significant associations with osteoporosis were also observed in the unadjusted model, Model I, and Model II (all *P* < .001).

**Table 5:** Multivariate logistic regression analysis.

Factor	Unadjusted model		Model I		Model II	
	OR (95% CI)	*P*	OR (95% CI)	*P*	OR (95% CI)	*P*
SIRI	4.383 (2.263–8.488)	*P* < .001	4.803 (2.356–9.791)	*P* < .001	5.841 (2.721–12.541)	*P*<.001
NLR	1.717 (1.290–2.286)	*P* < .001	1.774 (1.308–2.406)	*P* < .001	2.038 (1.410–2.945)	*P*<.001

Adjusted variables for Model I:age, exercise habits, smoking history, drinking history, and BMI. Adjusted variables for Model II: age, exercise habits, smoking history, drinking history, BMI, diabetes history, coronary heart disease history, chronic liver/kidney disease history, cerebrovascular disease history, hypertension history, number of pregnancies, age at menarche, TC, HDL-C, and LDL-C.

BMI = body mass index, HDL-C = high density lipoprotein cholesterol, LDL-C = low density lipoprotein cholesterol, NLR = neutrophil to lymphocyte ratio, SIRI = systemic inflammation response index, TC = total cholesterol.

### 3.5. Variable screening via LASSO regression

**Figure 1.**
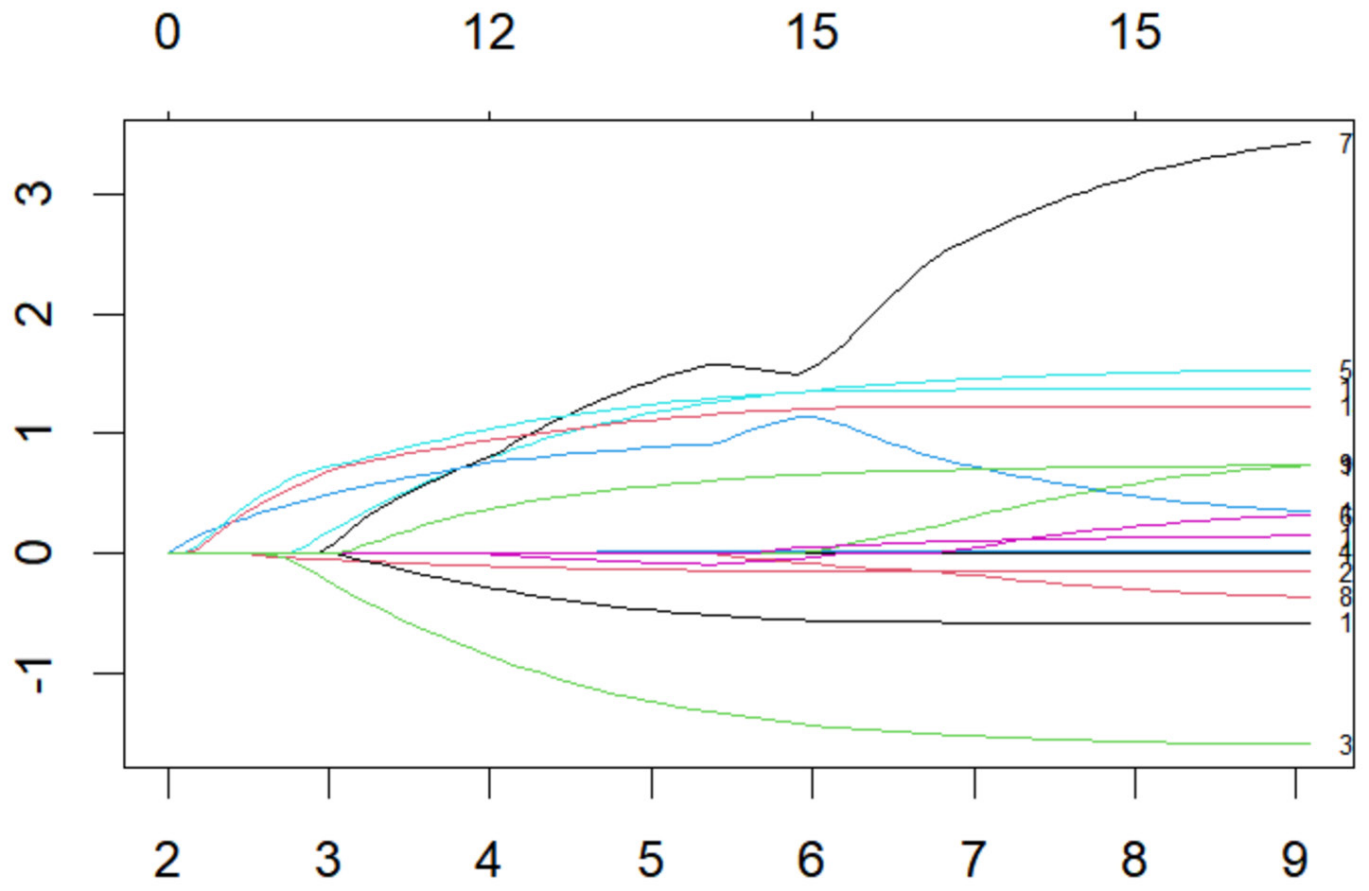
Review of coefficient path diagram.

**Figure 2.**
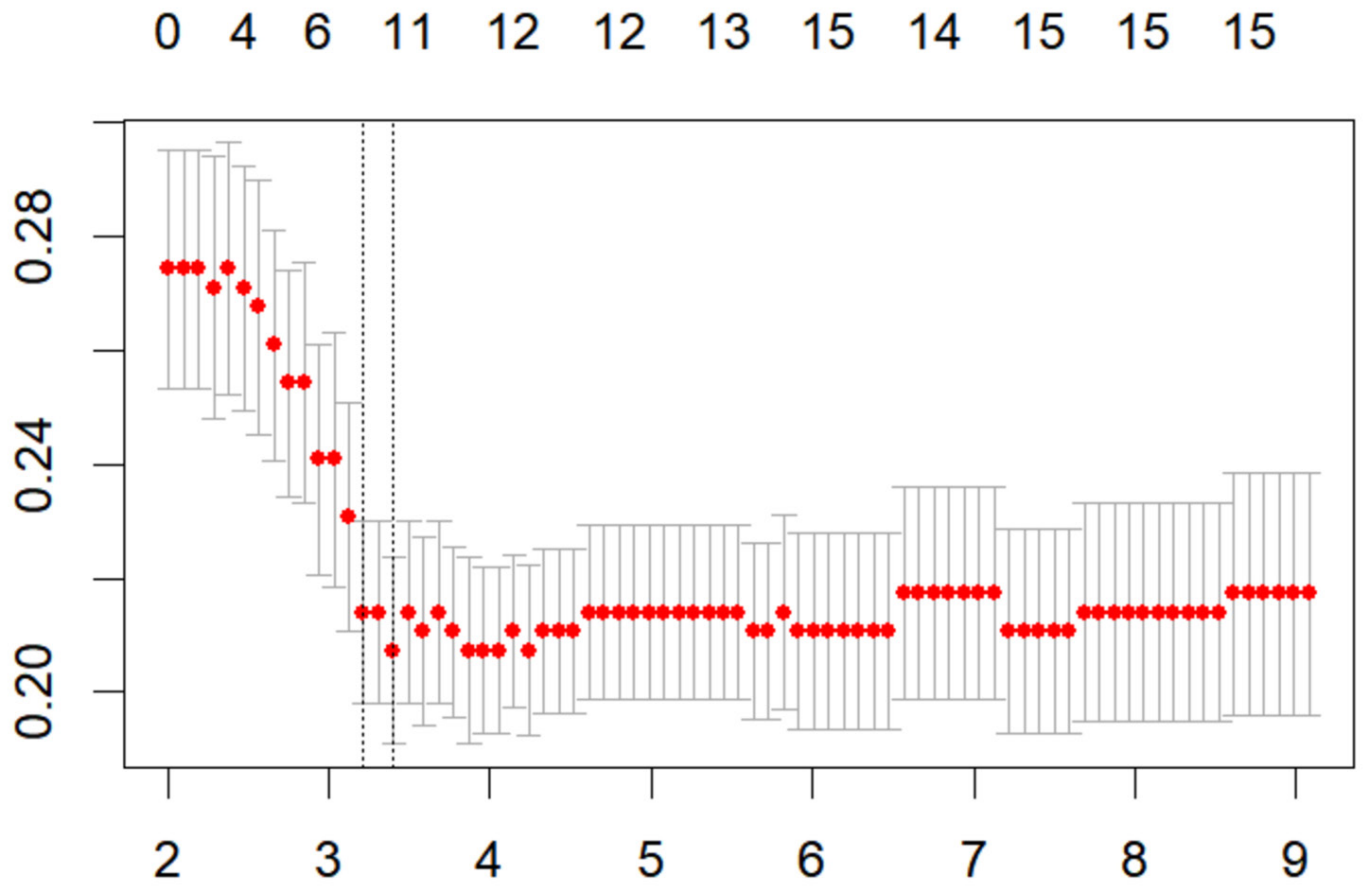
Cross-validation curve.

In addition to SIRI, LASSO regression was used to screen all variables from the univariate analysis to identify the factors most strongly associated with osteoporosis. The regression coefficient path diagram and cross-validation curve are shown in Figures 1 and 2, respectively. To avoid overfitting, 10-fold cross-validation was adopted to evaluate the generalization performance of the model. This strategy minimizes overfitting to the training data through multi-round, alternating validation set testing, ensuring the constructed model exhibits stable and reliable generalization performance when applied to external independent data. Considering factors including accessibility and clinical applicability, 4 key variables were ultimately selected: age, diabetes history, LDL-C, and frequent fall history.

### 3.6. Evaluation of predictive efficacy via ROC curve

**Figure 3.**
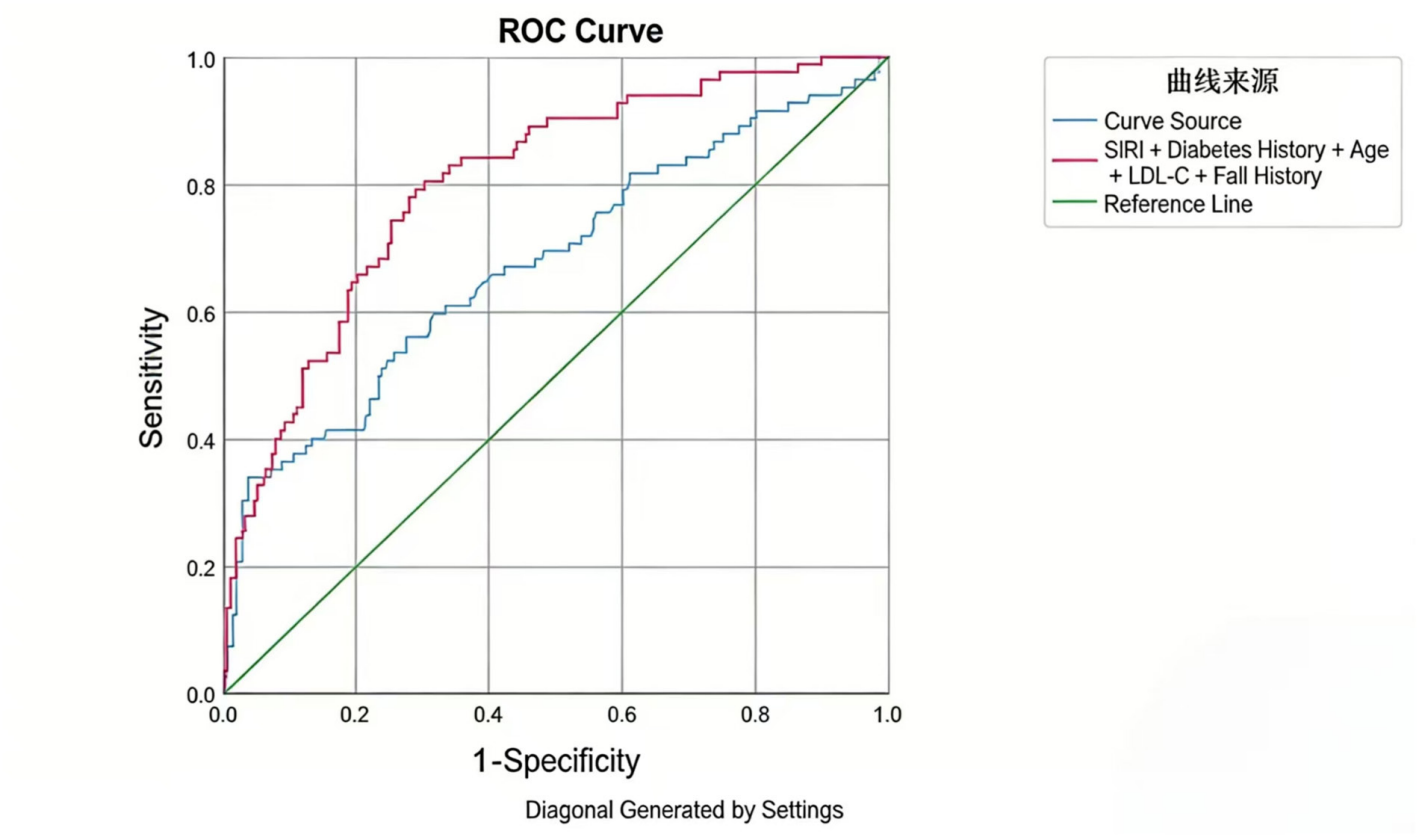
ROC curve monitoring the diagnostic value of SIRI and the combined model. LDL-C = low density lipoprotein cholesterol, ROC = receiver operating characteristic curve, SIRI = systemic inflammation response index

ROC curve analysis was performed to evaluate the clinical assessment value of SIRI and the combined model (SIRI, diabetes history, age, LDL-C, and history of frequent falls) for osteoporosis. As shown in Table 6 and Figure 3, the area under the curve (AUC) of SIRI for assessing the association with osteoporosis was 0.677 (95% CI: 0.604–0.749, *P* < .001), indicating moderate clinical reference value. Based on the Youden index (0.304), the optimal cutoff value of SIRI was determined to be 0.876, with a corresponding sensitivity of 34.1% and specificity of 96.3%.

**Table 6:** ROC curve evaluates the efficacy of SIRI in predicting the occurrence of OP.

Indicator	AUC	Youden Index	Cutoff value	Sensitivity (%)	Specificity (%)	95% CI
SIRI	0.677	0.304	0.876	34.1	96.3	0.604–0.749
Combined Model	0.802	0.503	0.212	79.3	71.0	0.747–0.857

The combined model includes SIRI + age + diabetes history + LDL-C + frequent fall history.

AUC = area under the curve, LDL-C = low density lipoprotein cholesterol, ROC = receiver operating characteristic curve, SIRI = systemic inflammation response index.

For the combined model incorporating SIRI, diabetes history, age, LDL-C, and history of frequent falls, the AUC was 0.802 (95% CI: 0.747–0.857, *P* < .001), with a Youden index of 0.503, optimal cutoff value of 0.212, sensitivity of 79.3%, and specificity of 71.0%. Visually, the ROC curve of the combined model lies above that of the single SIRI indicator and is farther from the reference line. This finding confirms that the combined model has enhanced clinical assessment utility for evaluating the association with osteoporosis.

## 4. Discussion

This study systematically explored the association between SIRI and osteoporosis in perimenopausal and postmenopausal women for the first time. The results demonstrated that SIRI is an independent risk factor for osteoporosis and exhibits moderate clinical reference significance. Combining SIRI with routine clinical indicators (age, diabetes history, LDL-C, and history of frequent falls) significantly improves clinical assessment utility, providing a convenient and reliable auxiliary indicator for the early identification of osteoporosis in this population.

The classic theory holds that osteoporosis is primarily caused by bone remodeling disorders resulting from estrogen deficiency, aging, or secondary factors (e.g., diseases and drugs).^[12]^ However, with the deepening understanding of the intricate “osteoimmunology” crosstalk between the skeletal and immune systems, the role of immune cells in osteoporosis has gained increasing attention.^[13]^ Various immune cells interact with osteoblasts and osteoclasts through direct cell-cell contact or paracrine mechanisms, thereby regulating bone metabolism.

Neutrophils play a protective role in bone formation during the early stage of bone healing. However, under conditions of estrogen deficiency, neutrophils become overactivated, releasing reactive oxygen species to promote osteoblast apoptosis and impairing bone homeostasis through RANKL signaling.^[10]^ monocytes, as precursor cells of multinucleated osteoclasts, directly affect bone metabolic balance and bone mass maintenance through their quantity and functional status.^[14]^ Imbalanced osteoclast formation can shift physiological bone remodeling to pathological remodeling, leading to the development of osteoporosis.^[15]^ A previous study reported that monocyte levels were higher in postmenopausal women with osteoporosis than in healthy women.^[16]^

In postmenopausal women, estrogen deficiency induces T-lymphocyte dysregulation and increases the circulating levels of inflammatory cytokines (e.g., interleukin-1β, IL-6, and TNF-α).^[17]^ These cytokines participate in RANK signaling and play a key role in regulating osteoclast formation and differentiation.^[18]^ Additionally, the number of B cells changes in postmenopausal women: a clinical study found that the number of CD19 + B lymphocytes was lower in postmenopausal female patients,^[19]^ while another study indicated that B cells exert a dual effect on bone metabolism-promoting osteoclast activation and differentiation while secreting TGF-β1 to inhibit pathogenic osteoclast formation.^[20]^ T cells have long been known to promote bone loss in inflammatory environments by secreting osteoclastogenic cytokines^[21]^; a study showed that T cells in postmenopausal osteoporosis patients with fractures are more likely to produce TNF-α.^[22]^ However, recent evidence suggests that T cells may also promote bone formation: the total number of T lymphocytes and absolute T-lymphocyte count were significantly lower in the osteoporosis group than in the non-osteoporosis group,^[23]^ and a positive correlation was observed between total lymphocyte and femoral BMD in healthy postmenopausal women.^[24]^

Novel inflammatory markers derived from routine hematological parameters (NLR, SII, and SIRI) have been shown to be associated with the occurrence and development of osteoporosis. NLR reflects the imbalance between pro-inflammatory neutrophil and anti-inflammatory lymphocyte. SII extends to the “inflammation-coagulation” interaction by incorporating platelet on the basis of NLR. SIRI, however, offers a unique advantage by comprehensively covering key immune cells (neutrophil, monocyte, and lymphocyte) involved in bone metabolism, thereby reflecting the balance between pro-inflammatory and anti-inflammatory cells.

In this study, SIRI showed a significant gradual increase among the normal bone mass group, osteopenia group, and osteoporosis group (*P* < .001) and maintained a stable negative correlation with BMD (*r* = −0.203, *P* < .001). Multivariate logistic regression analysis confirmed that SIRI remained an independent risk factor for osteoporosis after adjusting for 15 confounding variables (OR = 5.841, 95% CI: 2.721–12.541, *P* < .001). These results are consistent with a previous study by Yin et al, which reported a correlation between SIRI and bone turnover markers-indirectly supporting the potential mechanism by which SIRI affects osteoporosis occurrence through the regulation of bone metabolism.^[25]^

No significant association between SII and osteoporosis was observed in this study, which was an observational finding in a specific population. The underlying mechanism remains unclear, and the relevant analysis is only preliminary exploration without definitive inference, which needs to be verified by more studies.

From the preliminary analysis of clinical application scenarios, as a derivative index of routine blood test, SIRI theoretically has the potential advantages of low detection cost and easy clinical implementation. However, its actual application cost and popularization still need to be further evaluated based on large-sample health economics studies, and relevant quantitative analysis was not conducted in this study.

In this study, the AUC of SIRI for assessing the association with osteoporosis was 0.677, with a specificity of 96.3% at the optimal cutoff value (0.876). This high specificity indicates that using SIRI > 0.876 as an early warning threshold can accurately identify high-risk populations with a low missed diagnosis rate, making it suitable for large-scale preliminary screening. Furthermore, the combined model incorporating SIRI, age, diabetes history, LDL-C, and history of frequent falls yielded an AUC of 0.802, which significantly improving clinical assessment utility. These indicators are easily accessible in clinical practice, further enhancing the practicality of the combined model for OP identification.

This study has several limitations. First, it is a single-center retrospective study with a limited sample size, which may introduce selection bias. Given the inherent limitations of retrospective design, residual confounding cannot be completely ruled out: despite adjusting for 15 potential confounders (e.g., age, diabetes history, and lipid profiles), unmeasured or inadequately quantified factors may still affect the association between SIRI and osteoporosis. These factors include serum estrogen levels (a core mediator of postmenopausal bone loss), dietary calcium/vitamin D intake (key nutrients for bone metabolism), physical activity intensity beyond the current binary definition (moderate vs non-moderate), and frequency of hot flashes/sweating (symptoms linked to elevated cortisol and accelerated bone loss. Additionally, the findings may have limited generalizability to other populations due to the single-center Chinese cohort; variations in ethnic backgrounds, healthcare access, and lifestyle patterns could influence the correlation between inflammatory markers and osteoporosis. Second, no long-term follow-up was conducted, and dynamic validation data are lacking. In the future multi-center, prospective studies with larger sample sizes are needed to further verify the results and explore the underlying mechanism of SIRI in osteoporosis. In future studies, we recommend designing multi-center prospective cohorts with larger sample sizes, which can include subjects with a history of acute infections (with a standardized washout period of ≥ 3 months to ensure the resolution of acute inflammatory responses) to mitigate selection bias. Additionally, long-term follow-up of participants can help verify the clinical correlation significance of SIRI for incident osteoporosis and fracture outcomes, further enhancing the clinical applicability of the findings.

## 5. Conclusions

This study systematically explored the association between SIRI and osteoporosis in perimenopausal and postmenopausal women, and identified key risk and protective factors for the disease. The results showed that age, TC, monocyte, neutrophil, NLR, SIRI, diabetes history, frequent fall history, and fracture history are risk factors for osteoporosis in this population, while BMI, LDL-C, lymphocyte, and exercise habits act as protective factors. SIRI is negatively correlated with BMD and positively correlated with osteoporosis, with the risk of osteoporosis increasing alongside elevated SIRI levels within a certain range. Notably, SIRI is significantly associated with osteoporosis and provides moderate clinical reference significance for risk assessment, serving as a convenient auxiliary indicator for the early identification of high-risk individuals-especially valuable for perimenopausal and postmenopausal women facing estrogen deficiency-induced bone loss. Combining SIRI with age, diabetes history, LDL-C, and history of frequent falls further enhances clinical assessment utility for evaluating the association with osteoporosis. However, the current findings are based on a single-center retrospective study and require validation by prospective and external cohorts before any potential clinical application can be considered.

## Author contributions

**Data curation:** Xiaoning Wang.

**Formal analysis:** Xiaoning Wang.

**Funding acquisition:** Doufei Shi.

**Investigation:** Xiaoning Wang.

**Resources:** Doufei Shi.

**Supervision:** Doufei Shi.

**Writing – original draft:** Xiaoning Wang.

**Writing – review & editing:** Doufei Shi.
